# Differences in bleeding patterns and outcome after intracerebral hemorrhage due to vascular malformations

**DOI:** 10.1371/journal.pone.0217017

**Published:** 2019-05-23

**Authors:** Nazife Dinc, Sae-Yeon Won, Nina Brawanski, Michael Eibach, Johanna Quick-Weller, Jürgen Konczalla, Joachim Berkefeld, Volker Seifert, Gerhard Marquardt

**Affiliations:** 1 Department of Neurosurgery, Goethe University Hospital, Frankfurt, Germany; 2 Department of Neuroradiology, Goethe University Hospital, Frankfurt, Germany; Universitatsklinikum Freiburg, GERMANY

## Abstract

**Background:**

Atypical intracerebral hemorrhage is a common form of primary manifestation of vascular malformations.

**Objective:**

The aim of the present study is to determine clues to the cause of bleeding according to hemorrhage pattern (lobar, basal ganglia, infratentorial).

**Methods:**

We retrospectively evaluated 343 consecutive neurosurgical patients with intracerebral hemorrhage (ICH), who were admitted to our neurosurgical department between 2006 and 2016. The study cohort includes only neurosurgical patients. Patients who underwent treatment by neurologists are not represented in this study. We assessed location of hemorrhage, hematoma volumes to rule out differences and predicitve variables for final outcome.

**Results:**

In 171 cases (49.9%) vascular malformations, such as arteriovenous malformations (AVMs), cavernomas, dural fistulas and aneurysms were the cause of bleeding. 172 (50.1%) patients suffered from an intracerebral hemorrhage due to amyloid angiopathy or long standing hypertension. In patients with infratentorial hemorrhage a malformation was more frequently detected as in patients with supratentorial hemorrhage (36% vs. 16%, OR 2.9 [1.8;4.9], p<0.001). Among the malformations AVMs were most common (81%). Hematoma expansion was smaller in vascular malformation than non-malformation caused bleeding (24.1 cm^3^ vs. 64.8 cm^3^, OR 0.5 [0.4;0.7], p < 0.001,). In 6 (2.1%) cases diagnosis remained unclear. Final outcome was more favorable in patients with vascular malformations (63% vs. 12%, OR 12.8 [4.5;36.2], p<0.001).

**Conclusion:**

Localization and bleeding patterns are predictive factors for origin of the hemorrhage. These predictive factors should quickly lead to appropriate vascular diagnostic measures. However, due to the inclusion criteria the validity of the study is limited and multicentre studies with further testing in general ICH patients are required.

## Introduction

Intracerebral hemorrhage (ICH) harbors a mortality rate of up to 50% within the first 30 days.[1,2] Early and aggressive care has an enormous impact on outcome [3,4] and magnetic resonance angiography (MRA) and computed tomography angiography (CTA) are sensitive and required methods for identifying the cause of hemorrhage, such as arteriovenous malformations (AVMs), sinus thrombosis, tumors or moyamoya diseases.[5,6] Digital subtraction angiography (DSA) is considered to be most sensitive in verification of a suspected vascular malformation. Hypertensive blood pressure conditions or amyloid angiopathy are common causes for spontaneous intracerebral hemorrhage. Beside the high rate of mortality and morbidity with high lifetime costs, cognitive impairment and depression appear frequently in patients with spontaneous ICH.[7,8] Other causes for ICH are vascular pathologies, including AVMs. The annual bleeding risk of AVMs amounts 1% to 4% and mostly affects young patients.[2,9,10] Outcome after AVM hemorrhage seems to be more favorable in comparison to the outcome after sICH [11,12], depending on patient characteristics, bleeding patterns, hemodynamic features and hemorrhage size. The aim of our study is to align these predictive factors, which imply bleeding source.

## Methods

### Study design

Patients were recorded in our prospectively maintained database and analyzed retrospectively. The local ethical review board (ethic committee of the Goethe University Hospital Frankfurt, EK No 545/15) approved this study. Since the study design is retrospective, no written consent was provided and necessary by the patients.

**Fig 1:**
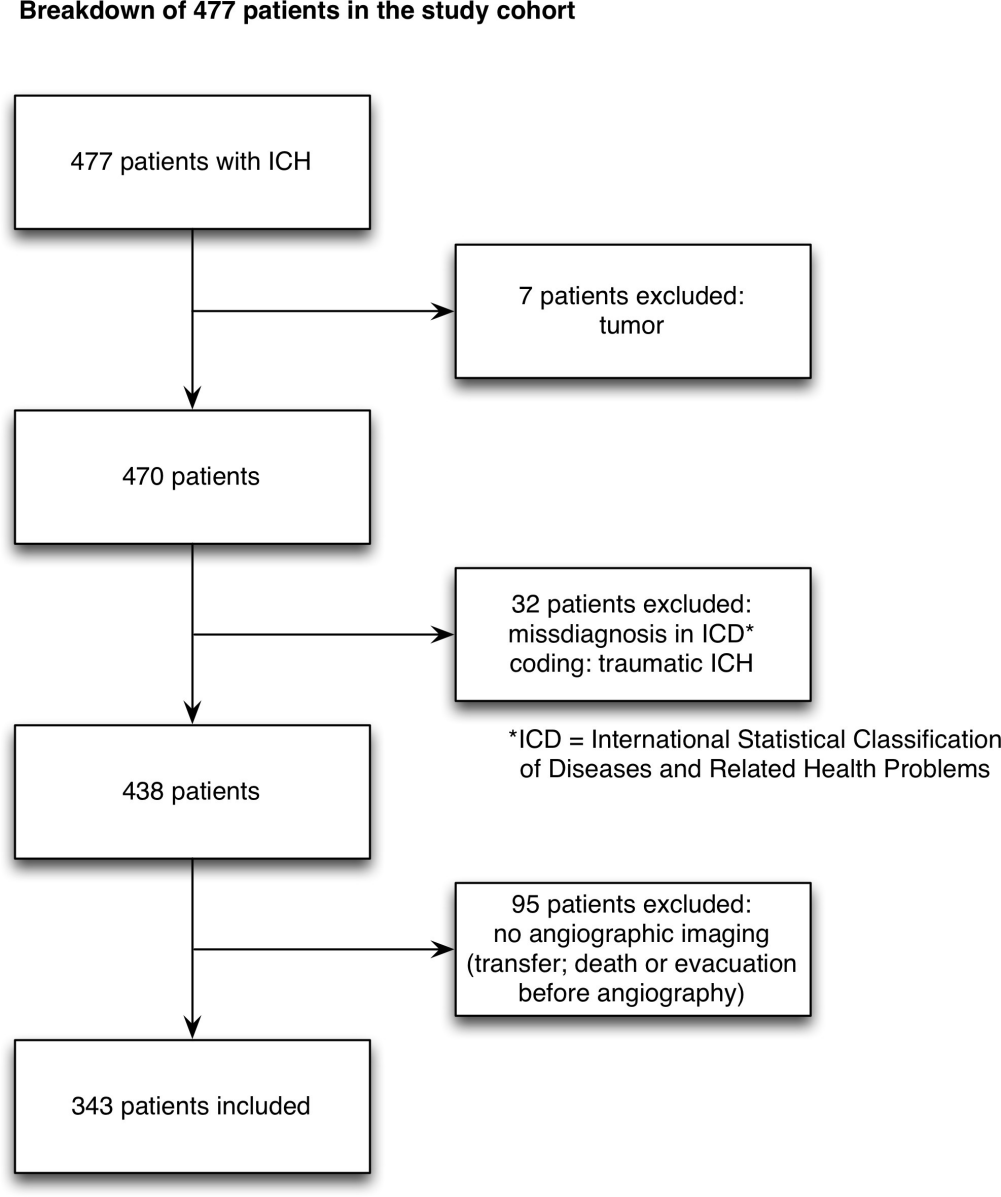
Flowchart for patients selection. Flowchart demonstrating the breakdown of 477 patients in the ICH database. 343 patients were enrolled for analysis.

All adult patients with intracerebral hemorrhage who were admitted to our department of neurosurgery between January 2006 and April 2016 were considered eligible. Patients with traumatic brain injury, subarachnoidal hemorrhage without a hematoma clot, hemorrhage due to a tumor or patients who did not receive angiographic imaging and patients who were admitted to the neurological department were not included in the study analyses. Therefore the study cohort is preselected in only including patients with ICH hemorrhage who were admitted to our neurosurgical department. A considerable number of patients who underwent treatment by neurologists are not represented in this study. MRA, CTA or DSA were required imaging methods for study inclusion criteria. Patient characteristics, such as neurological score at admission (based on the Glasgow Coma Scale: GCS [13]), gender, age, bleeding pattern (subarachnoid hemorrhage: SAH, intraventricular hemorrhage: IVH, intracerebral hemorrhage: ICH), hematoma volume, the use of antiplatelet drugs [AC], history of persistent arterial hypertension [HTN] were recorded and evaluated. Neurological score at admission was assigned to the following groups: good admission state (GCS 13–15) and poor admission state (GCS <7–12). Outcome was measured based on the modified Rankin Scale (mRS) at discharge and follow up visits after 6 months and stratified as mRS 0–2: favorable vs. mRS 3–6 unfavorable. Table 1 and Fig 1 represent inclusion and exclusion criterias and breakdown of the study cohort.

**Table 1:** The inclusion and exclusion criteria for the study cohort.

**Inclusion criteria**	
1	Age 18 years or older
2	Spontaneous intracerebral hemorrhage
3	Complete data (GCS, follow up/mRS)
4	Angiographic imaging (CTA, MRA, or DSA)
**Exclusion criteria**	
5	Traumatic hemorrhage (SDH, EDH, traumatic SAH)
6	Isolated SAH (aneurysmal SAH)
7	Incomplete data (GCS, follow up/mRS)
8	Previously known neoplasm or vascular malformation
9	Reliable angiographic imaging can not be performed (due to short-term hospital stay or urgent hematoma evacuation before angiographic imaging)

GCS = Glasgow coma scale; SDH = subdural hematoma; EDH = epidural hematoma, SAH = subarachnoid hemorrhage

### Radiological characteristics

Imaging was performed and interpreted by an experienced neuroradiologist. All study patients underwent angiographic imaging for diagnostic purpose. Atypical bleeding pattern were summarized as non-traumatic, non-aneurysmal, non-tumorous bleeding. Hematoma location was registered and classified into supratentorial, infratentorial, lobar and as basal ganglia hemorrhage (Table 2). The bleedings were divided into spontaneous hemorrhages without an underlying vascular malformation (sICH) and hemorrhages due to a vascular malformation (vmICH). Characteristics and type of vascular malformations (AVM, Cavernoma, dural arteriovenous fistula: dAVF, aneursyms) were evaluated. AVMs were graduated according to the Spetzler-Martin classification. The hematoma volume and patient age were evaluated to establish a cut off value of volume and age related to bleeding origin and poor outcome. The CT and MRA scans at admission were used for computer-assisted volumetric measurements. The method was used by an independent clinician. For computer-assisted hematoma volume measurement, the BrainLab elements software (BrainLab Germany Headquarters, Munich, Germany) was used.

**Table 2:** Patient characteristics and predictors for vmICH in univariate and multivariate analysis.

Factors	vmICH 171(%)	sICH 172(%)	Univariate analysis		Multivariate analysis	
			OR 95%CI	*p* value	OR 95% CI	*p* value
Female	82(48)	86(50)	NS	NS	NS	NS
Male	89(52)	86(50)	-	-	-	-
< 50 years (y)	92(54)	27(16)	6.3(3.8;10.4)	<0.001	4.1(1.8;9.2)	0.001
Mean age (y)	47	63	-	-	-	-
GCS (13–15)	119(70)	51(30)	4.8(2.9;7.9	<0.001	3.3(1.7;6.3)	<0.001
mRS 0–2**	108(63)	20(12)	11.7(6.6;20.9	<0.001	12.8(4.5;36.2)	<0.001
HTN*	43(25)	97(56)	0.3(0.2;0.4	<0.001	NS	NS
Anticoagulation	16(9)	58(34)	0.2(0.1;0.4)	<0.001	0.3 (0.1;0.7)	0.004
ICH location			2.7(1.6;4.6)	<0.001	3.1 (1.6;6.3)	<0.001
Supratentorial	109(64)	144(84)	0.3(0.2;0.6)	<0.001	-	-
Lobar	90(83)	104(72)	-	-	-	-
Deep ganglia	19(17)	40(28)	-	-	-	-
Infratentorial	62(36)	28(16)	2.9(1.8;4.9)	<0.001	-	-
IVH	65(38)	82(48)	NS	NS	NS	NS
Spot sign	22(13)	34(20)	NS	NS	NS	NS
Volume (cm^3^)	24.1	64.8	0.2 (0.1;0.3)	<0.001	0.5 (0.4;0.7)	0.001

* Chi square Test

**47 patients got lost to follow up, final outcome was available for 296 patients.

### Treatment

The treatment algorithm of intracerebral hemorrhage at our neurosurgical department is as follows: Usually, first admission setting is the closest facility or the emergency department. Patients get transfer to our neurosurgical department when intracerebral hemorrhage was already carried out via brain scan (CT or MRI). The primary objective in our neurosurgical setting is to check and support cardiovascular and ventilatory conditions. Secondary priorities include i.a. medical history, hemostatic abnormalities and medication. Based on interdisciplinary consensus medical management, particularly blood pressure management is carried out at the intensive care unit.[3,6] Continous cardiopulmonary monitoring including cycled automated blood pressure cuff, electrocardiograpic telemetry and pulse oxymetry is provided. Intra-arterial monitoring is considered in patients receiving intravenous vasoactive drugs. Since the study period amounts approximately 10 years, ICH guidelines changed within this period. The quality and availability of brain imaging has increased even in non-specialized facilities and patients were transferred more quickly to neurosurgical centers. In particular, due to the rising use of new anticoagulant agents [3] medical treament for normalization of hemostasis and the use of antidots changed. Furthermore according to the current guidelines for the management of ICH [14,15] urgent treatment such as decompressive craniectomy or hematoma evacuation is necessary in the presence of a space demanding hemorrhage, midline shift or loss of consciousness. Patients with basal ganglia hemorrhage or small hematoma volume in good clincal condition receive medical management. Patients with intraventricular hemorrhage receive an external ventricular drain. Secondary we performed a scan with angiogram (CTA/MRA) 4 to 6 hours later to capture hematoma expansion and detect bleeding origin. A catheter angiogram is considered if radiological and clinical suspicion of an underlying vascular cause is high or if vascular malformation is confirmed in CTA or MRA. When a vascular malformation is detected, the malformation will be treated by microsurgery, endovascular occlusion, radiosurgery, combined or conservatively, based on interdisciplinary consensus. In selected cases treatment of vascular malformations was performed after sufficient rehabilitation, e.g. 4 weeks after the ictus.

### Statistics

Statistical analysis was performed using the statistical software package SPSS (IBM SPSS Statistics for Windows, Version 22; Armonk, New York, USA. IBM Corp.) and the statistical software package BIAS (Version 11.08). Categorical variables were analyzed in contingency tables using the Fisher exact test, an unpaired t-test was used for parametric statistics. For univariate analysis statistical significance was set at p<0.05. Variables with a possible association with a vascular malformation (p<0.1) were entered into a forward stepwise multiple logistic regression analysis. The prognostic value of age and hematoma volume between the two groups was captured by calculating the area under the receiver operating characteristic (ROC) curve, which is a plot of sensitivity of predictions against 1-specificity of predictions. An area under the curve (AUC) of 0.5 indicates no discrimination between the two groups, and an area of 1.0 indicates perfect discrimination.[16]

## Results

### Patient characteristics

We evaluated 343 patients. The study group consisted of 168 women and 175 men with a mean age at first presentation of 55 years (range 19–89). Clinical data and radiological findings considering differences in bleeding origin are summarized in Table 2. In 172 patients a sICH could be detected, 171 patients suffered an ICH due a ruptured vascular malformation. 170 (49.6%) patients showed a good admission state (GCS 13–15) and 173 (50.4%) patients showed a severe ICH (GCS <7–12). Patients with a vascular malformation were younger on average (mean: 47 years vs. 63 years; OR 4.1 [1.8;9.2]; p<0.0001) and mostly in a better condition at admission than patients with sICH (OR 3.3 [1.7;6.3]; p<0.0001). Young age seems to be a statistical significant predictor for an underlying vascular malformation in patients with ICH. A cut off value of less than 55.5 years was calculated for patients with a vascular malformation (AUC = 0.7; 95%CI = [0.69;0.79], p<0.0001). Patients with a vascular malformation rarely had hypertension (OR 0.3 [0.2;0.4]; p<0.0001) and the use of antiplatelet drugs (OR 0.3 [0.1;0.7]; p<0.004) was less common.

### Radiological findings and Vascular Malformation features

Table 3 depicts the rate and classification of Vascular Malformations (VM). Arteriovenous malformations were the most common cause of vmICH. AVMs were detected in 138 (81%) patients, 70 (51%) AVMs were Spetzler Martin Grade I-II, 55 (40%) AVMs were Spetzler Martin Grade III and 13 (9%) AVMs were Spetzler Martin Grade IV-V. In 18 (10%) patients Cavernomas were found, in nine (5%) patients dural AVFs, and six (4%) patients were affected due to a ruptured aneurysm.

**Table 3:** Rate and classification of Vascular Malformations: Radiologic findings.

	No of patients N = 171 (%)
Cavernous malformation	18 (10)
AVM	138 (81)
Spetzler Martin I-II	70 (51)
Spetzler Martin III	55 (40)
Spetzler Martin IV-V	13 (9)
dAVF	9 (5)
Aneurysm	6 (4)
ICA right	2
AcomA	1
MCA right	1
ICA left	1
MCA left	1

ICA = internal carotid artery; AcomA = anterior communicating artery; MCA = middle cerebral artery

**Fig 2:**
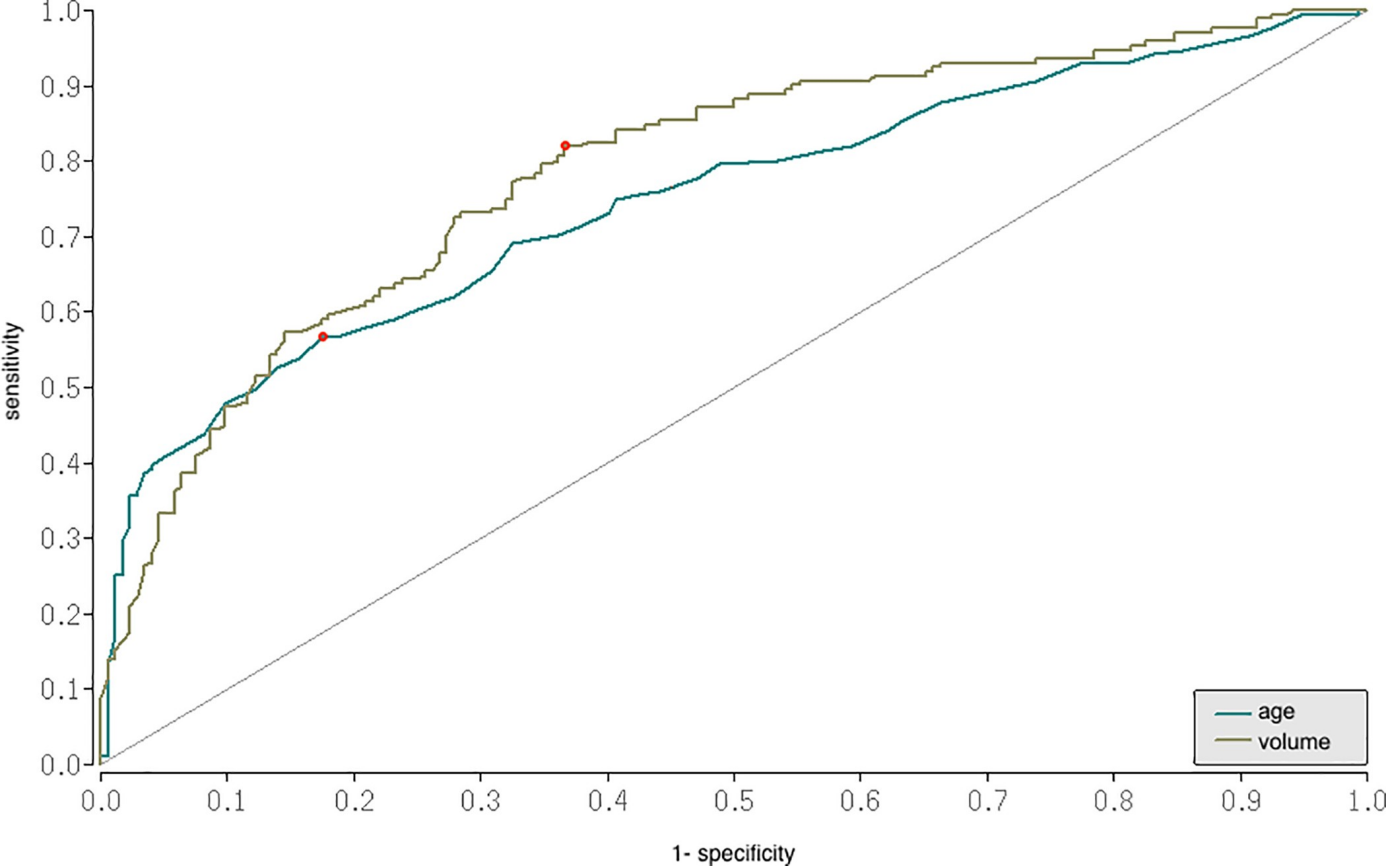
ROC curves for prediction of age and volume. Receiver operating curves for prediction of age and volume as diagnostic suitable variables for vascular malformations. Patients with vascular malformations were younger. The ROC curve for age yielded an AUC value of 0.7 (95%CI = 0.69;.079), p < .0001; p = 0.001) with a cut off value of 55.5 years. The ROC curve for volume yielded an AUC value of 0.8 (95%CI 0.53 [0.4;0.7]; p = 0.001) with a cut off value of 35.6 cm^3^. ROC = Receiver Operating Characteristic.

SICH was more common supratentorial and more often located in the basal ganglia than vmICH, whereas in infratentorial hemorrhage a vascular malformation was the most common cause of bleeding. (OR 3.1 [1.6;6.3]; p<0.0001). Hematoma volume was significant larger in sICH than in vmICH. The ROC curve (Fig 2) yielded an AUC of 0.8 (OR 0.53 [0.4;0.7]; p = 0.001) with a cut off value of 35.6 cm^3^ for bleeding origin.

### Urgent surgery

Urgent treatment such as decompressive craniectomy or hematoma evacuation was necessary in 139 patients (Table 4), mostly due to sICH 93(67%). Such a measure was required in 43 patients with vmICH 46(33%) (OR 3.2 [2.0; 5.0]; p< 0.001).

**Table 4:** Urgent surgery and mortality rate.

	sICH (%)	vmICH (%)	95% CI	*p* Value *
**Urgent surgery *N* = 139**	93 (67)	46 (33)	3.2 (2.0;5.0)	<0.001
**Overall mortality *N* = 39**	28 (72)	11 (28)	2.8 (1.4;5.9)	0.006

*Chi square

### Overall outcome and predictors for poor outcome in Multiple Logistic Regression analysis

**Fig 3:**
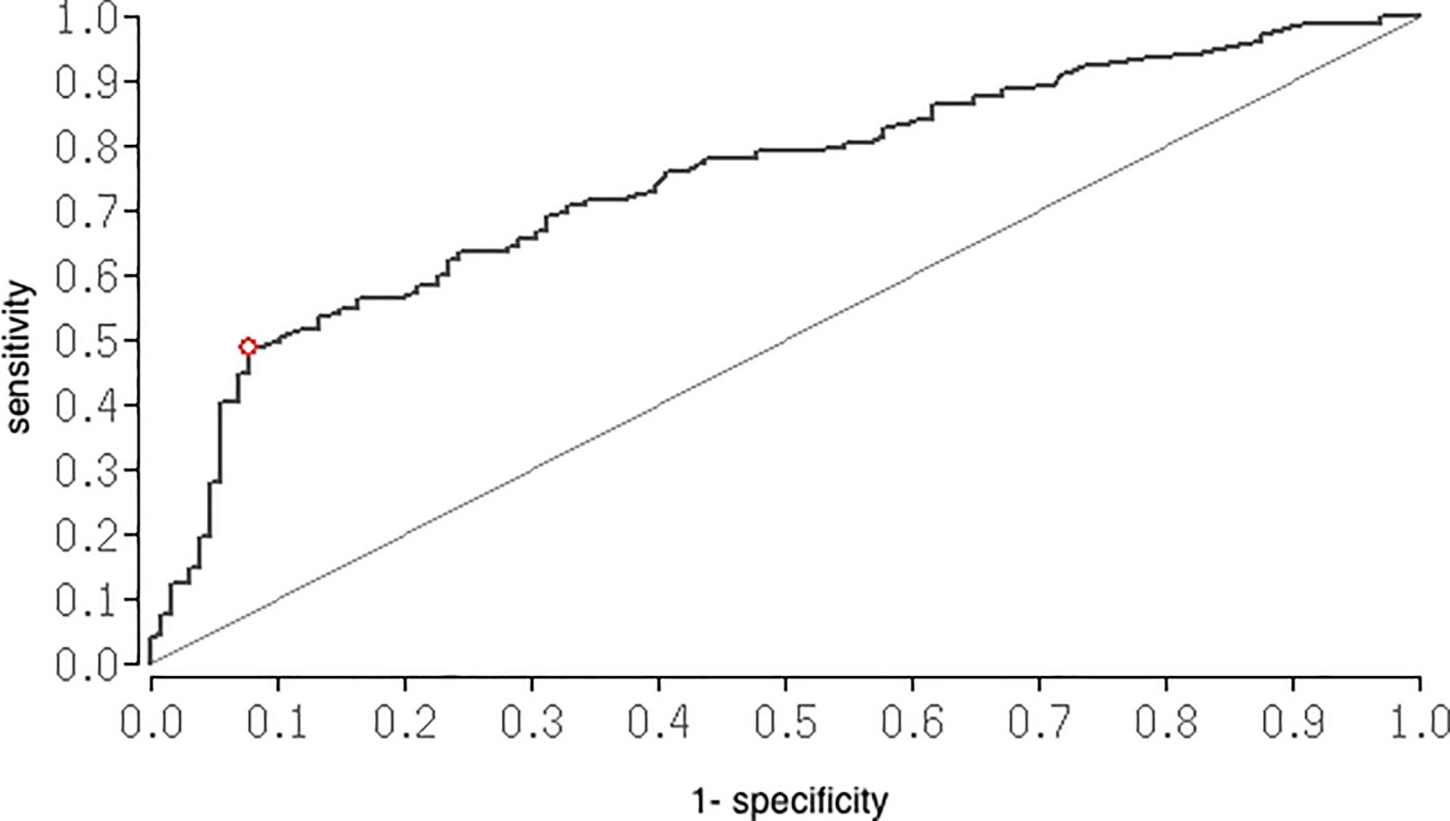
ROC for prediction of hemorrhage volume. Receiver operating curves for prediction of volume as diagnostic suitable variables for final outcome. The ROC curve yielded an AUC value of 0.8 (95%CI = 0.69; 0.80); p<0.0001 with a cut off value of 45.9cm^3^. ROC = Receiver Operating Characteristic.

Table 5 summarizes the rate of antiplatelet drugs, which were used by our study patients. Patients with antiplatelet drugs had a hematoma volume more than 30cm^3^ in 63.5% of cases compared with patients without antiplatelet drugs (40.9%). The use of antiplatelet drugs significantly led to a hematoma volume more than 30cm^3^ (OR 2.5 [1.48; 4.28]; p = 0.0006) and predicted primary outcome in univariate analyisis (OR 3.19[1.69; 6.00]; p = 0.0001). Patients with the use of Acetylsalicylacid and Clopidogrel had the highest hematoma volume on average. In ROC analysis, we identified a cut-off value of 45.9cm^3^ hematoma volume to predict outcome. The ROC curve (Fig 3) yielded an AUC of 0.8 (95%CI [0.69;0.80]; p<0.0001). Therefore we performed an univariate and multivariate logistic regression analysis to identify independent prognostic variables. In multivariate analysis poor admission state, hematoma volume and bleeding origin were independent predictors for poor outcome. Patients with vascular malformations showed more often a favorable outcome than patients with sICH (95% CI 9.3[4.5;19.5]; p < .0001). The overall mortality rate for the study patients was 11% (39 patients), mostly occurring in patients with sICH (71.8% vs. 28.2%; 95% CI 2.8 [1.4;5.9]; p = 0.006). Table 6 summarizes the predictive variables for outcome in univariate and multivariate analysis.

**Table 5:** Rate of antiplatelet drugs and association to hematoma volume.

	No N = 74 (%)	Mean hematoma volume (cm^3^)
Acetylsalicylacid +Clopidogrel	5 (7)	65.1
Acetylsalicylacid	29 (39)	62.8
Phenprocoumon	39 (53)	46.9
Rivaroxaban	1 (1)	42.0

**Table 6:** Univariate and multivariate analysis for primary favorable outcome.

**Factors**	**Univariate analysis**		**Multivariate analysis**	
	OR (95% CI)	*p* Value	OR (95% CI)	*p* Value
Age	0.3 (0.2;0.5)	<0.001	3.2 (1.9;5.6)	0.143
HTN	0.3 (0.2;0.5)	<0.001	1.6 (1.05;2.4)	0.965
Anticoagulation	3.19 (1.7;6.0)	<0.001	7.4 (2.9;18.7)	0.405
GCS	14.8 (7.7;28.4)	<0.001	13.16 (7.29;23.73)	<0.001
Vascular malformation	11.7(6.6;20.9)	<0.001	9.3 (4.5;19.5)	<0.001
Volume	0.2 (0.1;0.4)	<0.001	0.19 (0.11;0.31)	<0.001
IVH	2.1 (1.2;3.5)	0.01	0.6 (0.2;1.6)	NS
Urgent treatment	11.0 (5.9;20.4)	<0.001*	2.4(1.1;5.1)	0.02

*Chi square

## Discussion

### Outcome and prognosis in ICH

Age and clinical condition at ictus have the strongest association with further course and outcome after ICH.[17] These predictive variables are necessary to assess the risk for an underlying vascular malformation in intracerebral hemorrhage and to assess prognostic values. Many studies described scores to provide analysis of long-term prognosis for patients with intracerebral hemorrhage.[18,19] Furthermore the source of bleeding is one more and even crucial cause of poor or favorable outcome.[20] Therefore fast and appropriate diagnosis is required for impacting outcome positively. Bleeding pattern and patients´ medical history usually indicate bleeding source. Spontaneous ICH due to hypertension and cerebral amyloid angiopathy are common causes for ICH in elderly patients.[8,21] 40% of our patients presented hemorrhage due to an AVM. In 10% cavernous malformations, dural fistulae and aneurysms were found. Nevertheless, we must emphasize, that a considerable number of patients at our centre were treated by neurologists and were not eligible for this study, as mentioned in the Methods section. The risk of AVM hemorrhage depends on special AVM features, including AVM location and angioarchitecture [22,23] and the annual bleeding risk amounts approximately 1 to 4%.[10,24] While AVM rupture may lead to severe neurologic dysfunction or result in death,[25,26] many studies described differences in outcome after AVM hemorrhage and spontaneous hemorrhage and confirm a better outcome after AVM hemorrhage.[11,12,19]

### Bleeding pattern in VM hemorrhage

We found evident differences in patient characteristics, bleeding pattern and outcome between sICH and vmICH that require different counseling and treatment. Patients with vmICH were younger, healthier and less affected at ictus than patients with sICH. Infratentorial hemorrhage was more often associated with AVM hemorrhage than with sICH. Hematoma volume considerably affects patients outcome and a few studies discussed the high impact of hematoma volume on poor outcome.[27–29] Our study patients with vmICH had more often a small hematoma size and therefore were less affected. Several studies describe that AVM hematomas were mostly located within the nidus or in the venous side with sparing the healthy brain parenchyma which explains small hematoma size and better outcome.[30–32] The use of antiplatelet drugs was more common in patients with sICH and was significantly associated with a hematoma volume more than 30cm^3^. This was an additional finding and we recommend more studies with targeted research to evaluate the effect of antiplatelet drugs on hemorrhage volume. Regarding hematoma volume previous studies confirmed similar results.[33–35]

### Differences in treatment and outcome in sICH and vmICH

Furthermore there were significant differences in treatment timing and urgency. Patients with sICH were more often treated immediately after ictus and the overall mortality rate was higher. Patients with vmICH were mostly treated after recovery.

Beside age and the use of antiplatelet drugs, medical history, clinical condition at ictus, hemorrhage volume, location and in particular bleeding source were statistically evident predictors for final outcome.

### Generalizability and limitations

The knowledge of potential bleeding source, appropriated diagnostic methods and treatment time may improve young physicians´ practices in routine and emergent care and may lead to a high level of using the right and fast treatment methods.[21,36–38] Our findings support prior statements of lower case fatality after hemorrhage due to vascular pathologies, in particular due to AVM hemorrhage compared to outcome findings after sICH.[19,20] The mean age in our study is low compared to other ICH trials. One reason for this is the large number of patients with AVM hemorrhage. The younger age and therefore healthier baseline conditions in patients with vascular pathologies compared to patients with sICH are significant causes for a favorable outcome.

Therefore, it is important to make a reliable diagnosis based on appropriated diagnostics and to treat patients adequately in order to achieve a favorable outcome and for better advising patients and family members. In summary, finding differences in patient characteristics and bleeding pattern according to the bleeding origin is the main objective of our study in order to give clues for fast and reliable diagnostic. Secondarily we found differences in outcome in relation to the bleeding origin. Our study is limited by its retrospective design and single centre experience. Since the inclusion criteria required complete follow up data and angiographic imaging, patients without angiographic imaging could not be evaluated. A considerable number of patients undergo treatment by neurologists. Since routine diagnostic algorithm and treatment may differ, we included only patients who were admitted and treated neurosurgically. This is one reason why our cohort was preselected. This preselcetion is a major limitation and could introduce a selction bias.

## Conclusion

The major aim of our study was to identify predictive factors for underlying pathologies in ICH patients. ICH due to vascular malformation has some predictive features to detect the bleeding origin fast. Due to the inclusion criteria the validity of the study is limited and therefore more multicentre studies with further testing in general ICH patients in a prospective study design are required.
